# Correction: Post-Heparin LPL Activity Measurement Using VLDL As a Substrate: A New Robust Method for Routine Assessment of Plasma Triglyceride Lipolysis Defects

**DOI:** 10.1371/journal.pone.0099721

**Published:** 2014-06-02

**Authors:** The PLOS ONE Staff

## Notice of Republication

This article was republished on May 14, 2014, due to multiple instances of "±" being published as "+." The publisher apologizes for this error. Additionally, some symbols were missing from Figure 3. Please download the PDF again to view the corrected article and Figure 3. The originally published, uncorrected article and the republished, corrected article are provided here for reference.

## Supporting Information

File S1 Originally published, uncorrected article.
(PDF)

File S2 Republished, corrected article.
(PDF)
