# Supplementary material for: Post-Heparin LPL Activity Measurement Using VLDL As a Substrate: A New Robust Method for Routine Assessment of Plasma Triglyceride Lipolysis Defects
Source: PLoS One. 2014 May 2;9(5):e96482. doi: 10.1371/journal.pone.0096482 (PMC4008628; doi:10.1371/journal.pone.0096482)
Supplement: Data S1 — Optimization of substrate. (DOCX) [file pone.0096482.s001.docx]

**Supplemental data S1: Optimization of substrate**

Human VLDL substrate was prepared from 10 different serum samples. Lipids and lipoproteins of each VLDL substrate were assayed before use in order to check the composition of the substrate (apoB, C-II, C-III, VLDL cholesterol and triglycerides levels: see Supplemental data Table 1 below).

Composition of the VLDL substrate for LPL activity assay

Firstly, we aimed to get the largest amount of VLDL without chylomicrons from an optimal number of normolipidemic subjects (TG <1.75 mmol/l); a pool of 10 ml of serum from 10 patients produced about 500 µl of VLDL substrate (TG concentration about 7 mmol/l). Secondly we aimed to obtain similar lipoproteins profiles for the different pool of VLDL substrates. lipids and apolipoprotein concentrations obtained from 40 different pool of VLDL substrates were strongly homogeneous (see Supplemental data Table 1), especially for the triglycerides levels (CV 2.46%), close to 7 mmol/l; the other parameters were very reproducible and in accordance with normal VLDL profile as shown in Supplemental data Table 1; apoA1 and HDL cholesterol were undetectable.

Choice of substrate concentration

In order to establish the final TG concentration in the reagent mix (set at 1.8 mmol/l), we tested one control PHLA with 2 different VLDL substrates using 2 triglycerides concentrations in the mixture (TG 1.8 and 3.6 mmol/l). The kinetics of the reaction were linear only from 120 to 240 minutes for the mix with TG 3.6 mmol/l (see Supplemental data Figure 1); the PHLA activities of this control patient calculated in the linear range were very close to those obtained with TG at 1.8 mmol/L: respectively 23.7 and 19.8 µmol/l/min. The HL activities were not modified.

In order to verify the optimized TG concentration chosen for the mixture (1,8 mmol/l), we tested 2 samples of patients (PHLA activity 17.4 and 49.2 µmol/l/min) with 7 different concentrations of TG in the mixture (from 0.45 to 3.45 mmol/l) and calculated the PHLA activities from 60 to 180 minutes (see Supplemental data Figure 2); the same activity was found between 1.5 and 2.2 mmol/l of triglycerides in the different mixture and was slightly increased (+17, +24%) in the high TG concentration mixture ( > 2.7 mmol/l).
