# Supplementary figures and images for: Post-Heparin LPL Activity Measurement Using VLDL As a Substrate: A New Robust Method for Routine Assessment of Plasma Triglyceride Lipolysis Defects
Source: PLoS One. 2014 May 2;9(5):e96482. doi: 10.1371/journal.pone.0096482 (PMC4008628; doi:10.1371/journal.pone.0096482)

**Figure S1. Optimization of the assay: triglycerides concentration in the mixture.**


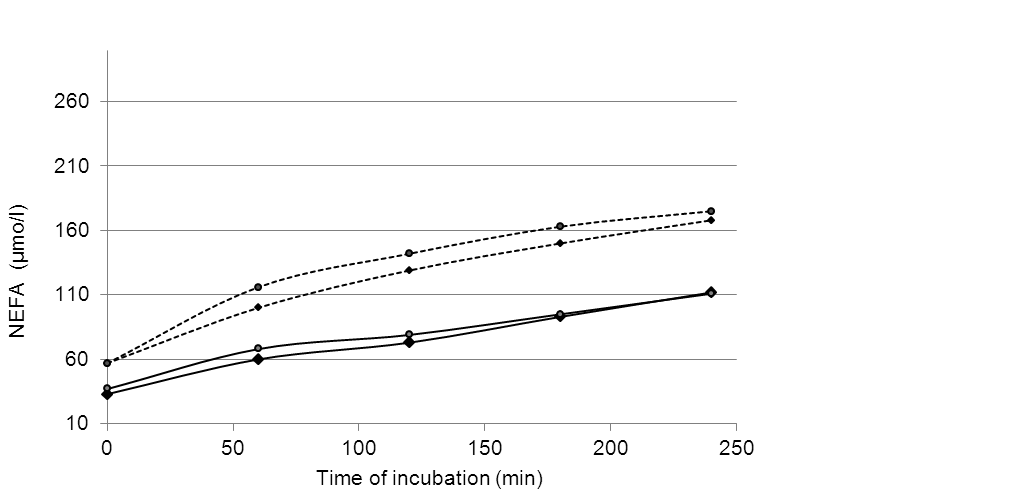

Supplement: Figure S1 — Optimization of the assay: triglycerides concentration in the mixture. Triglycerides concentration 25% (line); 50% (dotted line) Pool 23 (diamond); Pool 24 (circle) PHLA kinetics of 1 control plasma activity with 2 different VLDL substrates used at 2 TG concentrations (25 and 50%, i.e. TG 1.8 and 3.6 mmol/l). (DOC) [file pone.0096482.s002.doc]
