# Supplementary material for: Post-Heparin LPL Activity Measurement Using VLDL As a Substrate: A New Robust Method for Routine Assessment of Plasma Triglyceride Lipolysis Defects
Source: PLoS One. 2014 May 2;9(5):e96482. doi: 10.1371/journal.pone.0096482 (PMC4008628; doi:10.1371/journal.pone.0096482)
Supplement: Figure S2 — PHLA measured with different triglycerides concentration substrate in the reaction. Patient 1 PHLA = 17.4 µmol/l/min (black line); Patient 2 PHLA = 49.2 µmol/l/min (grey line) 100% of activity was fixed with triglycerides in the mixture at 1.8 mmol/l. (DOC) [file pone.0096482.s003.doc]

**Figure S2. Post Heparin Lipase Activity measured according to different triglyceride concentrations in the the reaction substrate.**
