# Supplementary material for: Post-Heparin LPL Activity Measurement Using VLDL As a Substrate: A New Robust Method for Routine Assessment of Plasma Triglyceride Lipolysis Defects
Source: PLoS One. 2014 May 2;9(5):e96482. doi: 10.1371/journal.pone.0096482 (PMC4008628; doi:10.1371/journal.pone.0096482)
Supplement: Table S1 — Lipids and apoproteins of the VLDL substrates (n = 40 different pools of VLDL). (DOC) [file pone.0096482.s004.doc]

**Table S1. Lipids and apoproteins of the VLDL substrates (n=40 different pools of VLDL).**

| **Colonne1** | **units** | **mean** | **SD** | **CV** | **range** | **mean level in the MIX** |
| --- | --- | --- | --- | --- | --- | --- |
| **Triglycerides** | mmol/l | 7.29 | 0.18 | 2.46% | 6.95 - 7,63 | 1.82 |
| **Cholesterol** | mmol/l | 5.12 | 0.34 | 4.70% | 4.49 - 6,20 | 1.28 |
| **VLDLC/TG** |  | 0.31 | 0.02 | 6.40% | 0.27 - 0,36 |  |
| **apoB** | g/l | 0.68 | 0.04 | 5.90% | 0.62 - 0,80 | 0.17 |
| **apoC-II** | mg/l | 142 | 13 | 9.10% | 122 - 167 | 35.5 |
| **apoC-III** | mg/l | 302 | 25 | 8.30% | 258 - 345 | 75.5 |
| **C-II/C-III** |  | 0.47 | 0.03 | 6.40% | 0.39 - 0.54 |  |
