# Supplementary material for: Post-Heparin LPL Activity Measurement Using VLDL As a Substrate: A New Robust Method for Routine Assessment of Plasma Triglyceride Lipolysis Defects
Source: PLoS One. 2014 May 2;9(5):e96482. doi: 10.1371/journal.pone.0096482 (PMC4008628; doi:10.1371/journal.pone.0096482)
Supplement: Table S2 — Genetic variants identified in candidate genes in TVHTG patients. (DOC) [file pone.0096482.s005.doc]

**Table S2. Genetic variants identified in candidate genes in TVHTG patients.**

| **Gene** | **Exon** | **Variation** | **Position** | **Missense mutations Polyphen/SIFT predictions** | **Truncated protein** | **References** | **Hom** | **Cpd Het** | **Het** |
| --- | --- | --- | --- | --- | --- | --- | --- | --- | --- |
| *LPL* | 2 | p.W91X | c.272G>A | - | yes | SNPdb rs118204070 |  |  | 1 |
|  | 3 | p.A98T | c.292G>A | Probably damaging/tolerated |  | Chan 2002 [1] |  |  | 1 |
|  | 4 | p.P200LfsX7^£^ | c.599del | - | yes | Pruneta-Deloche, 2005 [2] |  |  | 1 |
|  | 5 | p.G215E | c.644G>A | Probably damaging/deleterious | yes | Reina 1992 [3] | 1 |  |  |
|  | 5 | p.V227A | c.680T>C |  |  | Maruyama 2004 [4] |  |  | 1 |
|  | 5 | p.V227G | c.680T>G | Probably damaging/Deleterious |  | **New mutation** |  | 1 |  |
|  | 5 | p.I252T | c.755T>C | Probably damaging/deleterious | yes | Henderson 1998 [5] |  | 1 |  |
|  | 6 | p.Y315ter^£^ | c.945T>A | - | yes | Causeret, 2001 [6] | 1 |  |  |
| *APOA5* | 3 | p.Q97X^£^ | c.283C>T | - | yes | Charrière 2009 [7] |  |  | 4 |
|  | 3 | p.Y110LfsX158 | c.327dup | - | yes | **New mutation** |  |  | 1 |
|  | 3 | p.Q139X^£^ | c.415C>T | - | yes | Marçais 2005 [8] |  |  | 3 |
|  | 3 | p.P215L | c.644C>T | Probably damaging/deleterious |  | SNPdb rs76753536 |  |  | 1 |
|  | 3 | p.L253P | c.758T>C | Probably damaging/deleterious |  | Mendoza-Barbera 2013 [9] |  |  | 1 |
|  | 3 | p.Q295X | c.883C>T | - | yes | **New mutation** |  |  | 1 |
|  | 3 | pR343C | c.1027C>T | Probably damaging/deleterious |  | **New mutation** | 1 |  |  |
| *GPIHBP1* | 3 | p.C89F*^,£^ | c.266G>T | Probably damaging/deleterious |  | Charrière 2011 [10] |  | 1 |  |
|  | 4 | p.G175R^£^ | c.532G>C | Probably damaging/tolerated |  | Charrière 2011 [10] | 1 |  |  |
| *APOC2* |  | pK41T | c.122A>C | Benigne / deleterious |  | [Hegele (1991)](http://www.ncbi.nlm.nih.gov/sites/entrez?cmd=Retrieve&db=PubMed&list_uids=1782747&dopt=Abstract) [11]  SNPdb rs120074114 |  |  | 1 |
| *APOE* |  | p.Q264R | c.791A>G | Probably damaging/deleterious |  | **New mutation** |  |  | 1 |

*In silico* analysis of the mutations was performed with Alamut v2.0 (Interactive Software), Polyphen (<http://genetics.bwh.harvard.edu/pph/>) and SIFT (http://sift.jcvi.org/www/SIFT _aligned_ seqs_submit.html).

^£^ mutation already published by our team ; * compound heterozygous patient: p.C89F associated with a large deletion of the second allele

1. Chan LY, Lam CW, Mak YT, Tomlinson B, Tsang MW, et al. (2002) Genotype-phenotype studies of six novel LPL mutations in Chinese patients with hypertriglyceridemia. Hum Mutat 20: 232-233.
2. Pruneta-Deloche V, Marcais C, Perrot L, Sassolas A, Delay M, et al. (2005) Combination of circulating antilipoprotein lipase (Anti-LPL) antibody and heterozygous S172 fsX179 mutation of LPL gene leading to chronic hyperchylomicronemia. J Clin Endocrinol Metab 90: 3995-3998.
3. Reina M, Brunzell JD and Deeb SS (1992) Molecular basis of familial chylomicronemia: mutations in the lipoprotein lipase and apolipoprotein C-II genes. J Lipid Res 33: 1823-1832.
4. Maruyama T, Yamashita S, Matsuzawa Y, Bujo H, Takahashi K, et al. (2004) Mutations in Japanese subjects with primary hyperlipidemia--results from the Research Committee of the Ministry of Health and Welfare of Japan since 1996. J Atheroscler Thromb 11: 131-145.
5. Henderson HE, Bijvoet SM, Mannens MA, Bruin T, Erkelens DW, et al. (1998) Ile225Thr loop mutation in the lipoprotein lipase (LPL) gene is a de novo event. Am J Med Genet 78: 313-316.
6. Causeret AS, Souillet AL, Marcais C, Prunetta V, Lachaux A, et al. (2001) [Familial hyperchylomicronemia with a new mutation of the lipoprotein lipase gene]. Ann Dermatol Venereol 128: 1343-1345.
7. Charriere S, Cugnet C, Guitard M, Bernard S, Groisne L, et al. (2009) Modulation of phenotypic expression of APOA5 Q97X and L242P mutations. Atherosclerosis 207: 150-156.
8. Marcais C, Verges B, Charriere S, Pruneta V, Merlin M, et al. (2005) Apoa5 Q139X truncation predisposes to late-onset hyperchylomicronemia due to lipoprotein lipase impairment. J Clin Invest 115: 2862-2869.
9. Mendoza-Barbera E, Julve J, Nilsson SK, Lookene A, Martin-Campos JM, et al. (2013) Structural and functional analysis of APOA5 mutations identified in patients with severe hypertriglyceridemia. J Lipid Res 54: 649-661.
10. Charriere S, Peretti N, Bernard S, Di Filippo M, Sassolas A, et al. (2011) GPIHBP1 C89F neomutation and hydrophobic C-terminal domain G175R mutation in two pedigrees with severe hyperchylomicronemia. J Clin Endocrinol Metab 96: E1675-1679.
11. Hegele RA, Connelly PW, Maguire GF, Huff MW, Leiter L, et al. (1991) An apolipoprotein CII mutation, CIILys19----Thr' identified in patients with hyperlipidemia. Dis Markers 9: 73-80.
