# Supplementary material for: Correction: Post-Heparin LPL Activity Measurement Using VLDL As a Substrate: A New Robust Method for Routine Assessment of Plasma Triglyceride Lipolysis Defects
Source: PLoS One. 2014 Jun 2;9(6):e99721. doi: 10.1371/journal.pone.0099721 (PMC4041855; doi:10.1371/journal.pone.0099721)
Supplement: File S2 — Republished, corrected article. (PDF) [file pone.0099721.s002.pdf]

Post-Heparin LPL Activity Measurement Using VLDL As a Substrate: A New Robust Method for Routine Assessment of Plasma Triglyceride Lipolysis Defects

Mathilde Di Filippo[1,2]*, Christophe Marçais[2,3], Sybil Charrière[2,4], Oriane Marmontel[1], Martine Broyer[1], Mireille Delay[3], Micheline Merlin[3], Axel Nollace[1], René Valéro[5], Michel Lagarde[2], Valérie Pruneta-Deloche[2], Philippe Moulin[2,4], Agnès Sassolas[1,2]

1 UF Dyslipidémies Cardiobiologie, Département de Biochimie et Biologie Moléculaire, Centre de Biologie et de Pathologie Est, Laboratoire de Biologie Médicale Multi Sites, Hospices Civils de Lyon, Bron, France, **2** INSERM U1060, INSA de Lyon, INRA U1235, Univ Lyon-1, Université de Lyon, Villeurbanne, Oullins, France, **3** Laboratoire de Biochimie spécialisée, Centre de Biologie Sud, Centre Hospitalier Lyon-Sud, Laboratoire de Biologie Médicale Multi Sites, Hospices Civils de Lyon, Pierre-Bénite, France, **4** Fédération d'endocrinologie, maladies métaboliques, diabète et nutrition, Hôpital Louis Pradel, Hospices Civils de Lyon, Bron, France, **5** Département de Nutrition, Maladies Métaboliques, Endocrinologie, APHM, Hôpital de la Timone, Aix-Marseille Université, UMR_S 1062, UMR_A1260, Marseille, France

Abstract

Background: Determination of lipoprotein lipase (LPL) activity is important for hyperchylomicronemia diagnosis, but remains both unreliable and cumbersome with current methods. Consequently by using human VLDL as substrate we developed a new LPL assay which does not require sonication, radioactive or fluorescent particles.

Methods: Post-heparin plasma was added to the VLDL substrate prepared by ultracentrifugation of heat inactivated normolipidemic human serums, diluted in buffer, pH 8.15. Following incubation at 37°c, the NEFA (non esterified fatty acids) produced were assayed hourly for 4 hours. LPL activity was expressed as µmol/l/min after subtraction of hepatic lipase (HL) activity, obtained following LPL inhibition with NaCl 1.5 mmol/l. Molecular analysis of *LPL*, *GPIHBP1*, *APOA5*, *APOC2*, *APOE* genes was available for 62 patients.

Results: Our method was reproducible (coefficient of variation (CV): intra-assay 5.6%, inter-assay 7.1%), and tightly correlated with the conventional radiolabelled triolein emulsion method (n = 26, r = 0.88). Normal values were established at 34.8±12.8 µmol/l/min (mean±SD) from 20 control subjects. LPL activities obtained from 71 patients with documented history of major hypertriglyceridemia showed a trimodal distribution. Among the 11 patients with a very low LPL activity (< 10 µmol/l/min), 5 were homozygous or compound heterozygous for *LPL* or *GPIHBP1* deleterious mutations, 3 were compound heterozygous for *APOA5* deleterious mutations and the p.S19W *APOA5* susceptibility variant, and 2 were free of any mutations in the usual candidate genes. No homozygous gene alteration in *LPL*, *GPIHBP1* and *APOC2* genes was found in any of the patients with LPL activity >10 µmol/l/min.

Conclusion: This new reproducible method is a valuable tool for routine diagnosis and reliably identifies LPL activity defects.

Citation: Di Filippo M, Marçais C, Charrière S, Marmontel O, Broyer M, et al. (2014) Post-Heparin LPL Activity Measurement Using VLDL As a Substrate: A New Robust Method for Routine Assessment of Plasma Triglyceride Lipolysis Defects. PLoS ONE 9(5): e96482. doi:10.1371/journal.pone.0096482

Editor: Paulo Lee Ho, Instituto Butantan, Brazil

Received November 8, 2013; **Accepted** April 7, 2014; **Published** May 2, 2014

Copyright: © 2014 Di Filippo et al. This is an open-access article distributed under the terms of the Creative Commons Attribution License, which permits unrestricted use, distribution, and reproduction in any medium, provided the original author and source are credited.

Funding: The authors have no support or funding to report.

Competing Interests: The authors have declared that no competing interests exist.

* E-mail: mathilde.di-filippo@chu-lyon.fr

Introduction

Lipoprotein lipase (LPL) (glycerol ester hydrolase, EC 3.1.1.34) plays a crucial role in the metabolism of very low density lipoproteins (VLDL) and chylomicrons [1,2]. A large pool of this enzyme bound to heparin sulfate proteoglycans and glycosylphosphatidylinositol-anchored high density lipoproteins binding protein 1 (GPIHBP1) through ionic linkage [3,4], is releasable into plasma following the intravenous injection of heparin. Familial LPL deficiency is a rare autosomal recessive disorder which is characterized by primary hyperchylomicronemia due to homozygous or compound heterozygous mutations of *LPL* gene [5] as well as to homozygous mutations of *APOC2*,*GPIHBP1*, *APOA5* or *LMF1* genes [6–8]. Additionally, sporadic forms of severe hypertriglyceridemia commonly result from complex interactions between environmental and genetic factors leading to various degrees of LPL deficiency [6]. Consequently, LPL activity measurement remains primordial in order to decipher the mechanisms underlying determinants of these heterogeneous phenotypes and bring irreplaceable information in case of negative or unavailable molecular diagnosis.

However, the LPL activity assay remains difficult. Methods using commercial triglyceride (TG) emulsions as a substrate are hampered by a high NEFA concentration at the basal state and a lack of specificity due to the presence of mono and diglycerides [9,10]. The use of trioleyl substrates with the incorporation of

radioactive tracers (^{3}H) by sonication under strictly controlled conditions by Nilsson-Ehle et al published in 1972 [11], was a major improvement. However, this time-consuming assay needs to be performed in triplicate in order to minimize the intra-assay error [12–15]. Despite several improvements, most of the current methods using radiolabelled (^{3}H or ^{14}C) substrates cannot be run on automats and require cumbersome strategies to inhibit hepatic lipase activity. Some chromophoric [16–20] or fluorescent [21] substrates have been used; however the preparation of these substrates is problematic, the derivatives were unstable and the method not sensitive enough [21]. In a recent method, Basu D et al [15] used a commercially fluorescent substrate (EnzChek), esterified by BODIPY-C12 at the *sn-1* position of glycerol, which was stable and solubilised with a specific detergent (Zwittergent). Although highly appealing, this method has not been tested in human post-heparin plasmas.

Therefore, we decided to set up a new reliable method sensitive enough to allow human post-heparin LPL activity measurement in routine conditions without requiring sonication, nor use of a fluorescent or radioactive substrate. Seeking reliability, we preferred to choose a natural substrate composed of pooled human VLDL, thereby also providing the optimal amount of human apoC-II.

Materials and Methods

Ethics Statement

Clinical investigations have been conducted according to the principles expressed in the Declaration of Helsinki. Informed written consent was obtained from the control subjects and the patients before DNA sampling and heparin injection. The study protocol was approved by our institutional ethical committee (Comité d'Ethique du CHU de Lyon).

Patients

Twenty adults control subjects (9 men, 11 women, mean age 52±17 years) were recruited in order to determine normal post-heparin LPL activity (without history of hypertriglyceridemia or diabetes). 71 hypertriglyceridemic patients with documented history of type V dyslipidemia (TVHTG) were assessed with the new method (Plasma TG concentration (TG) >15 mmol/l or familial history of hypertriglyceridemia with TG>10 mmol/l). Patients characteristics are summarized in Table 1.

Fasting venous blood samples were drawn into EDTA tubes on ice before (T0) and 10 min (T10) after a 50 U/kg intravenous heparin injection. The tubes were immediately centrifuged at 4°C and plasma was frozen at −80°C.

VLDL substrate preparation

VLDL substrate was used for the LPL assay: 20 ml of human serum were pooled from set of 10 human serum samples (TG 0.9–1.75 mmol/l, HDL-cholesterol >0.9 mmol/l, total cholesterol 3.2–5.5 mmol/l). VLDL from the pool were isolated in 1.2 ml polycarbonate tubes (Beckman) by preparative ultracentrifugation (d = 1.006 kg/l) using a Sorval Discovery M150 SE ultracentrifuge (Sorval S140 AT 017 Rotor: 140000 RPM (1042000 max) for 70 minutes). Floating VLDL were collected, stored at + 4° and used within 48 h; 360 µl of VLDL substrate at a final TG concentration of 7.3 mmol/l was used for both total post heparin and hepatic lipase activity assay. Before use, the VLDL substrate was heated at 56°C for one hour in order to inactivate any residual endogenous lipase activity.

Table 1. Subjects features.

lane		n	Age year	Sex %of men	BMI kg/m^2	Smoker % of smoker	Diabetes %
0	Controls	20	51.7 (22–76)	55	NA	NA	0
1	HTG without mutation group: no mutation in *LPL, GPIHBP1, APOA5, APOC2* was identified	15	45.3 (13–61)	73	27.1 (21.9–34.7)	20	45
2	HTG minor polymorphism group: *APOA5* p.S19W or SNP1 heterozygote, *APOA5* p.S19W homozygote, *APOC2* p.K41T heterozygote	20	48.4 (23–80)	80	27.6 (17.8–34.0)	42	50
3	HTG mild mutation group: homozygous mutation of *APOA5*, heterozygous mutation of *APOA5, LPL, GPIHBP1*	7	42.3 (36–50)	71	26.0 (20.3–32.0)	60	14
4	HTG mild mutation group and polymorphism: heterozygous mutation of *APOA5* (p.Q97X, p.Q295X, p.P215L, p.L253P) associated with S19W	8	48.4 (32–64)	50	27.5 (21.7–40.0)	33	38
5	HTG major mutation group: homozygous mutation of *LPL* or *GPIHBP1*	5	23.3 (5–41)	60	20.1 (19.1–21.0)	33	33
6	Patients with anti-LPL antibodies and low LPL activity	3	54.0 (35–70)	67	22.1 (21.8–22.3)	NA	NA

n effective of the group.
NA not available.
Mean (range) or %.
doi:10.1371/journal.pone.0096482.t001

Michaelis constant

The Michaelis constant (K_m) was determined as the inverse of the Lineweaver-Burk line intercept using 6 different VLDL substrates with final TG concentration ranging from 0.80 to 6.80 mmol/l.

LPL activity assay

The inactivated VLDL substrate and the mixture for the enzymatic reaction were kept on ice until the start of the reaction at 37°C.

Total post-heparin lipase activity (PHLA) (A): 180 µl of VLDL substrate was mixed with 540 µL of buffer A (Tris 50 mmol/l, $MgCl_2$ 3 mmol/l, $CaCl_2$ 1.5 mmol/l, dodecylbetain (laurylbetain Ramidus AB, Ideon, SE-223 70 Lund, Sweden) detergent 0.03%, pH 8.15) leading to a 1.8 mmol/l final TG concentration; 10 µl of post-heparin plasma sample (T10) were added and mixed; 120 µl were distributed into 5 polycarbonate tubes. At the beginning of the enzymatic reaction, one tube was kept on ice (basal NEFA level), 4 tubes were incubated in a 37° shaking water-bath and each hour, one tube was removed and kept on ice. The NEFA concentration was assayed on the 5 tubes by an enzymatic method in duplicate (Wako kit, NEFA-HR(2)) on a Pentra 400 Roche instrument. Calculation of the enzymatic activity was made in the linear part of the curve (see results) between 60 and 180 minutes. The PHLA activity was expressed in µmol/l/min of released NEFA.

Hepatic Lipase (HL) activity (B): the assay was conducted with the same procedure, but with 20 µl of post-heparin plasma sample and buffer A was substituted by buffer B (Tris 50 mmol/l, NaCl 2 mmol/l, dodecylbetain detergent 0.03%, pH 8.15) in order to inhibit the LPL activity; the final NaCl concentration in the mix was 1.5 mmol/l.

LPL activity was obtained by the subtraction of hepatic lipase activity, measured with buffer B from total activity obtained with buffer A and expressed as µmol/l/min of produced NEFA. The enzymatic reaction was controlled using, as the external standard, a sample of a frozen pool of PHLA plasmas obtained from 8 control subjects.

LPL assay comparison with the radiolabelled method

A radiolabelled ^{14}C-triolein emulsion was used as previously described [22]. The assays were performed in triplicate.

Molecular diagnosis

Following the extraction of genomic DNA from blood (Nucleon Bac3, GE Healthcare, Chalfont St. Giles, UK), the encoding regions, and the flanking intronic junctions of *LPL, APOA5, APOC2 GPIHBP1* and *APOE* genes were PCR amplified as previously reported [23-26]. The amplicons were directly sequenced with the BigDye Terminator v3.1 Cycle Sequencing Kit on an ABI PRISM 3730 DNA sequencer (Applied Biosystems, Foster City, USA).

In silico analyses of the mutations were performed with Alamut v2.0 (Interactive Software), Polyphen (http://genetics.bwh.harvard.edu/pph/) and SIFT (http://sift.jcvi.org/www/SIFT_aligned_ seqs_submit.html).

Statistical analysis

Statistical analyses were performed using SPSS 17.0 software. T-test or non-parametric Mann Whitney tests were performed in order to compare mean/median of LPL activities between groups. Shapiro-Wilk test was used to assess the normality of the distribution of the post heparin LPL activities. p-values (two sided) less than 5% were considered significant. Linear regressions were performed to determine the correlation between LPL activity and age or the conventional radiolabelled method.

Results

Optimization of substrate

(see Supplemental data S1 and Figure S1)

First, we verified that similar lipoprotein profiles were obtained from different VLDL substrate pools (n = 40, the coefficient of variation (CV) was <5% for cholesterol and TG concentrations and <10% for apoB, C-II and C-III) (see Table S1). Second, the mean K_m of the reaction was lower than 1.5 mmol/l (mean 1.42 mmol/l). In order to determine the optimal TG concentration chosen for the mixture, we tested 2 patient samples (PHLA activity 17.4 and 49.2 µmol/l/min) with 7 different TG concentrations in the mixture (ranging from 0.45 to 3.45 mmol/l); the activity was decreased (−44, −67%) with low TG levels (< 0.90 mmol/l) and slightly increased (+17, +24%) with high TG concentration (> 2.7 mmol/l) in the mixture (see Figure S2). Consequently, we established the optimal final concentration of TG in the assay mixture at 1.8 mmol/l.

Kinetics

Using the final substrate concentration set at TG = 1.8 mmol/l, the enzymatic reaction was found to be linear from 60 to 240 min of incubation, independently of the PHLA level, as shown in Figure 1A. Hepatic Lipase (HL) activity was low in these assay conditions and remained similarly low despite both a pH increase to 9.2 (optimal pH for HL) and a sample volume increased to 20 µl.

In order to increase the accuracy of the LPL assay by optimizing the amount of released NEFA, the reaction time was set to 3 hours since both enzymatic PHLA and HL reactions were linear from 60 to 240 min; PHLA activity was calculated between 60 and 180 min, in order to avoid a possible loss of linearity after 180 min.

Limit of detection

The minimum level of detectable LPL activity was determined through two methods: first, we performed the assay using 6 pre-heparin control plasmas and second, with 5 inactivated (56°C one hour) control post-heparin plasmas. We discovered that the minimal detectable LPL activity (mean+3SD) was 2.7 µmol/l/min for the pre-heparin plasmas (from VLDL-bound LPL or other circulating lipases) and 1.68 µmol/l/min for the heat inactivated post-heparin plasmas.

Linearity

A strict linearity of the LPL assay was observed between 0–70 µmol/l/h, as determined by the increase or decrease of the plasma sample volume from 6 patients with normal or high LPL activity (from 20 to 100 µmol/l/min); the kinetics of the enzymatic reactions were linear with any sample volume (3 to 20 µl) (Figure 1B). Consequently, the high (>70 µmol/l/min) and low (<10 µmol/l/h) LPL activities should be reassayed with a decreased (5 µl) or increased (20 µl) volume of assay sample respectively.

Imprecision

The coefficient of variation (CV) of the intra-assay reproducibility was 5.6% for an LPL activity of 31.5 µmol/l/min (SD: 1.8 µmol/l/min).

The inter-assay reproducibility was studied by including a single sample from a PHLA frozen control pool in 16 consecutive series

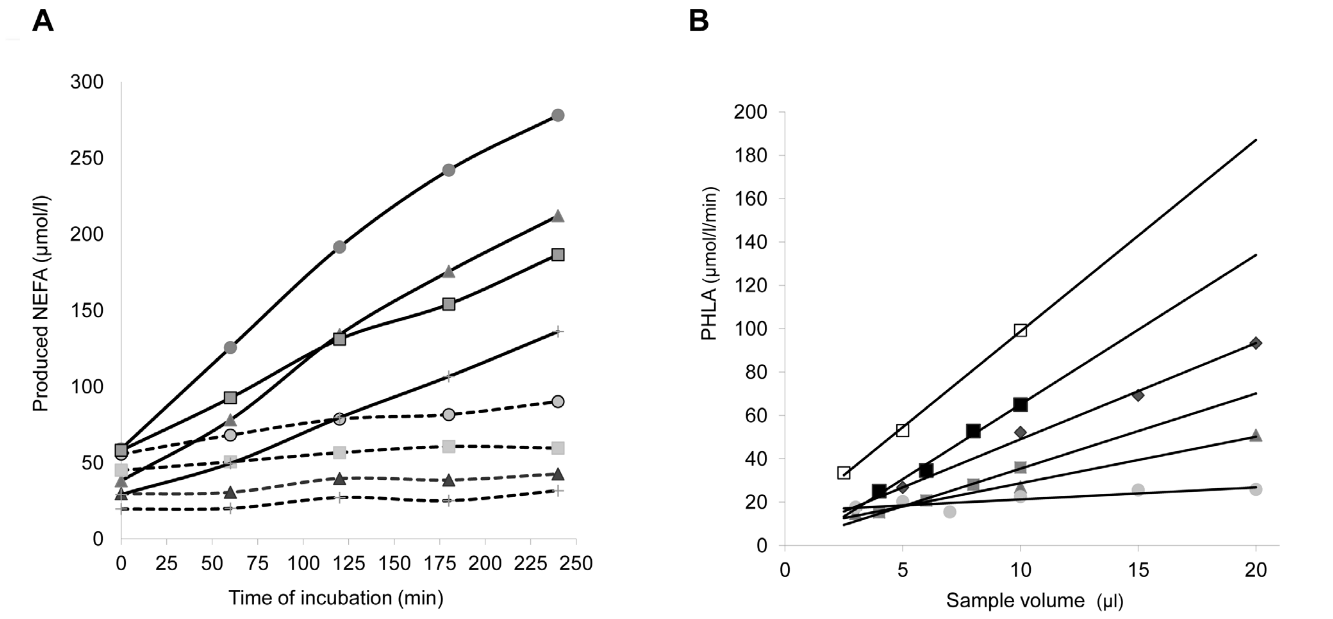

Figure 1. Kinetics and linearity. Figure 1A. PHLA and HL kinetics in 3 patients. LPL+HL (line); HL (dotted line); Patient 1 (triangle); Patient 2 (circle); Patient 3 (square); Patient 4 (vertical line). Figure 1B. Linearity test. Patient 1(black diamond); Patient 2 (white square); Patient 3 (grey triangle); Patient 4 (black square); Patient 5 (grey square); Patient 6 (grey circle).
doi:10.1371/journal.pone.0096482.g001

of independent LPL determinations; the CV was 7.1% (mean: 29.1±2.1 µmol/l/min). This sample allows an internal quality control of each assay.

Comparison with conventional radiolabelled method

26 post-heparin plasma LPL-HL activities obtained from hypertriglyceridemic patients with history of documented major hyperchylomicronemia were assayed for LPL activity by both methods (our new method versus the conventional ^{14}C triolein method). LPL activities showed a strong positive correlation (r = 0.88, p<0.001) (Figure 2A).

LPL activity in controls and TVHTG patients

Normal values of LPL activity were established at 34.8± 12.8 µmol/l/min (mean±SD; extreme values 10.6 to 62.2) in 20 control subjects (Figure 2B); the LPL activity was unchanged according to age (no correlation between LPL activity and age, r = 0.063) and gender: mean 30.9±11.7 µmol/l/min and 39.6+/− 13.0 µmol/l/min in 11 females and 9 males respectively (p = 0.261).

Interestingly, LPL activities from the 71 TVHTG patients were widely distributed (activity range 0 to 103 µmol/l/min; mean: 30.5±18.2 µmol/l/min) with evidence for a non normal distribution (p<0.01) (Figure 2B). 11 patients had a low LPL activity (<

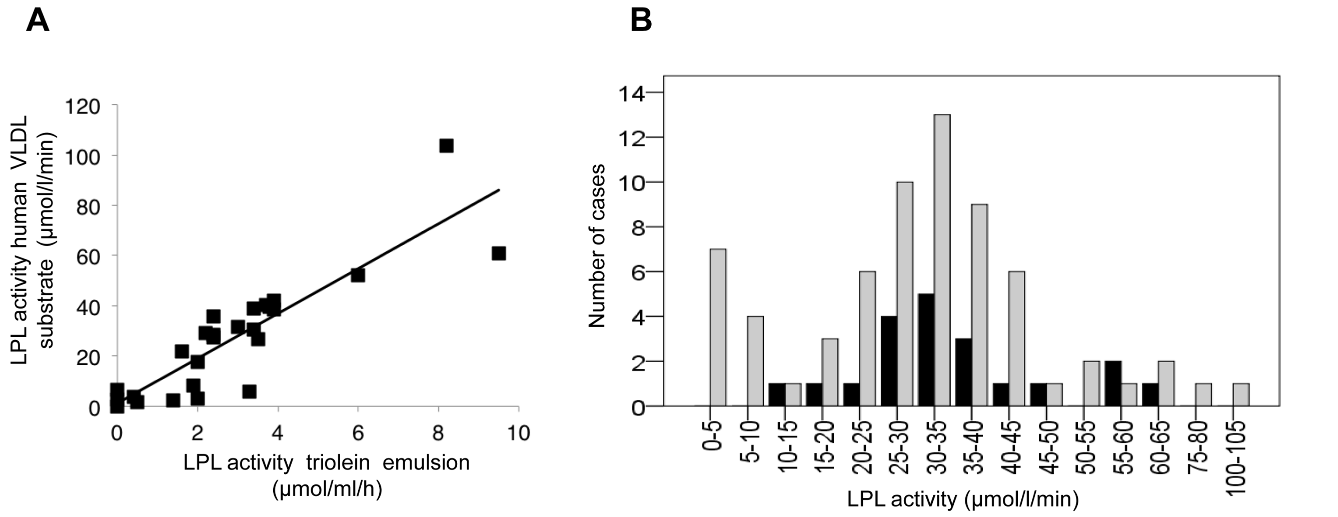

Figure 2. Correlation between LPL activity assays in TVHTG and controls patients. Figure 2A. Correlation with conventional method (n = 26, r = 0.88, p<0.001). y = 8.93× + 1.21; R^2 = 0.77. Figure 2B. Distribution of LPL activity in TVHTG patients and controls subjects. TVHTG patients (grey square); controls (black square).
doi:10.1371/journal.pone.0096482.g002

10 µmol/l/min (mean ± SD: 3.6 ± 2.4 µmol/l/min)) and the LPL activities in the other patients with LPL between 10 and 55 µmol/l/min were normally distributed around the median of this group (p = 0.97, median: 31.6 µmol/l/min, mean: 32.1± 8.6, n = 51). A group of 6 TVHTG patients had high LPL activity despite hypertriglyceridemia.

LPL activity and molecular diagnosis in hypertriglyceridemic patients with TVHTG

Among the 71 patients with history of major hypertriglyceridemia included, molecular assessment was available for 62 patients; all of which were examined for *LPL* gene mutations. 58 patients had a molecular diagnosis for additional candidate genes involved in the regulation of LPL activity (*APOA5*, *GPIHBP1*, *APOC2*, *APOE*). 5 new mutations were found: p.V227G in *LPL* gene, p.Y110LfsX158, p.Q295X and p.R343C in *APOA5* gene and p.Q246R in *APOE* gene (See Table S2).

Mutations in *LPL*, *GPIHBP1*, *APOA5* or *APOC2* genes were not found in 15/58 (26%) TVHTG patients (Figure 3, lane 1). In this group, most patients (13/15) presented normal LPL activity whereas 2 patients had both a very low LPL activity (1.6 and 3.7 µmol/l/min) and a clear familial history of hyperchylomicronemia. 1 of these 2 patients was found to be a carrier of p.Q246R a new missense heterozygous *APOE* gene mutation.

LPL activity was found in the reference interval in the 20/58 patients who harboured only *APOA5* or *APOC2* susceptibility polymorphisms (*APOA5* p.S19W, *APOA5* haplotype 2 or *APOC2* p.K41T) (lane2). LPL activity was also found in the reference interval in the 7/58 patients who had either heterozygous *LPL* or *APOA5* deleterious mutation as well as in a single patient with a homozygous deleterious *APOA5* missense mutation (lane 3).

Conversely all the 5 homozygous or compound heterozygous patients with deleterious *LPL* or *GPIHBP1* mutations had a drastically reduced LPL activity (lane 5 and see Table S2).

We considered a subgroup of TVHTG patients (8/58) defined as compound heterozygotes for a deleterious *APOA5* mutation and, on the second allele, a susceptibility polymorphism (either *APOA5*-haplotype 2 or *APOA5*-haplotye 3 (p.S19W)) (lane 4). 3 of these patients presented a low LPL activity < 10 µmol/l/min while 5 had a normal LPL activity.

3 patients had LPL auto-antibodies, only 1/3 displayed a low LPL activity (lane 6) at the time of blood drawing (3.1 µmol/l/min): this patient was also heterozygous for the *LPL* p.P200LfsX7 frame shift mutation and was previously reported with low LPL activity using the triolein emulsion conventional assay [27].

Overall, drastically reduced LPL activity (<10 µmol/l/min) was discovered in 11 TVHTG patients (TG mean ± SD: 20.5±27.0 mmol/l): 5 of which were identified with obvious causal genotypes in either *LPL* or *GPIHBP1* genes and 4 of which had genotypes most likely to contribute to their hyperchylomicronemia. Additionally, 2 pediatric patients with family history of dominant hypertriglyceridemia had a clear lipolysis defect although there was no causal genotype yet identified in candidate genes.

Compared to patient with low LPL activity (<10 µmol/l/min), the 47 genotyped (TVHTG) patients with LPL activity over 10 µmol/l/min had milder hypertriglyceridemia (8.9±8.0 mmol/l p<0.01). In this group, no correlation between LPL activity and

Figure 3

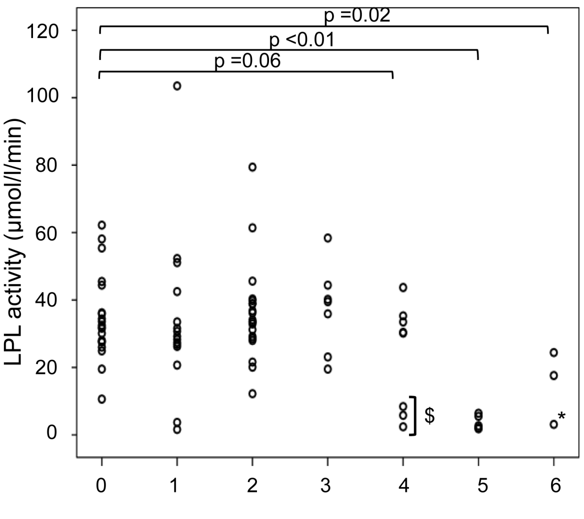

lane		n	TG: mean (range) (mmol/l)£	LPL activity: (µmol/l/min)
0	control	20	1.3 (0.6-2.8)	34.8+/-12.8
1	TVHTG no mutation group: no mutation in *LPL*, *GPIHBP1*, *APOA5*, *APOC2*	15	7.8 (1.0-27.2)	33.9+/-23.8
2	TV HTG minor polymorphism group: *APOA5* p.S19W or SNP1 heterozygote, *APOA5* p.S19W homozygote, *APOC2* p.K41T heterozygote	20	9.0 (1.1-31.5)	36.1+/-14.4
3	TVHTG mild mutation group: homozygous mutation of *APOA5,* heterozygous mutation of *APOA5*, *LPL*, *GPIHBP1*	7	8.3 (2.9-19.6)	37.3+/-13.1
4	HTG mild mutation and polymorphism: group heterozygous mutation of *APOA5* (p.Q97X, p.Q295X, p.P215L, p.L253P) associated with S19W	8	20.4 (3.5-99.9)	23.7+/-15.7
5	HTG major mutation group: homozygous mutation of *LPL* or *GPIHBP1*	5	12.3 (5.3-22.4)	3.7+/-2.1
6	patients with anti-LPL antibodies and low LPL activity	3	11.1 (8.3-13.9)	15.0+/-10.9

Figure 3. LPL activity and molecular diagnosis. $ 3 diabetic compound heterozygous patients ([p.Q139X];[p.S19W polymorphism]); * patient with anti-LPL antibodies and heterozygous mutation of *LPL* gene; £ Triglyceridemia determined the day of LPL activity measurement.
doi:10.1371/journal.pone.0096482.g003

plasma TG concentration was found (r = 0.14). None of these patients had either homozygous or combined heterozygous deleterious mutations in *LPL*, *APOC2*, or *GPIHBP1*.

Discussion

We propose a robust, very reproducible and convenient method to determine LPL activity in human post-heparin plasma. The absence of radiolabelled emulsion prevents the requirement of unreliable sonication under stringent conditions and avoid the use of costly and problematic reagent. Human VLDL constitute the natural substrate of LPL with an optimal composition in apoC-II and apoC-III similar to the concentrations found in normal plasma. The use of a pool of 10 plasmas obtained from control subjects provides a reproducible composition and lipolysis ability. Since fresh VLDL substrate for each LPL activity assay was needed, care was taken to control each assay with a pool of PHLA control plasmas. The dilution of the VLDL substrate was performed in a TRIS buffer in order to provide the necessary ions, optimal pH and suitable detergent conditions for an optimal reaction. A pH of 8.15 was considered as the optimal pH for the LPL activity [9,17,28–30]. The low HL activity found with our new method could be due to the fact that VLDL is not the optimal substrate for HL [31].

Since the kinetics of PHLA with a human VLDL substrate was not yet documented, we ascertained the linearity and the Michaelis constant (K_m) of the enzymatic reaction. The K_m found using human VLDL was in the range of several K_m reported using artificial TG emulsions (0.1 to 2.5 mmol/l) [9,16,32]. We chose to work with a substrate concentration of 1.8 mmol/l; within these conditions, the upper linearity of the LPL activity is sufficient (70 µmol/l/min); however the highest LPL activity level needs a decrease of sample volume while the lowest LPL activity may need an increase of volume. The use of a substrate TG concentration set at 1.8 mmol/l offers many advantages: firstly, the substrate can be prepared from normal serums; secondly, the required amount of TG VLDL is low.

The robustness of the method is optimal. The inter-assay reproducibility (CV 7.1%) is lower than those of most conventional triolein methods (mean CV 11.6%, range 5–25%) reported from 11 different methods by Henriksen [12–15,17,29,33,34]. The correlation with a radiolabelled triolein method was tight (r = 0.88), similar to that obtained by Imamura (dioleyl substrate versus labelled triolein, r = 0.79) (p = 0.21) [35]. Surprisingly, the other published methods with non-labelled soluble substrates did not provide any correlation with the conventional radiolabelled emulsion method [15–19].

Post-heparin LPL activity level in our normal controls (34.8 ± 12.8 µmol/l/min) was in the low range of those reported by the conventional method [24,36]. However, a huge heterogeneity is found amongst the published methods. The differences in normal LPL activity levels could be due to the use of different substrates, various doses of injected heparin (from 10 to 100 UI/kg body weight), apoC-II concentrations, pH of the reaction mixture, concentration of albumin, and method of inhibition of LPL and HL. All of these discrepancies were well described by Henriksen et al. who reported 11 published LPL activity assays, the reference values ranging from 25 to 362 mU/ml [34].

Our method provides a convenient tool to identify patients with a major defect in LPL activity. All patients with homozygous or compound heterozygous mutations of *LPL* and *GPIHBP1* were below the cut off point of 10 µmol/l/min, which is in agreement with previous reports for *LPL* and *GPIHBP1* genes mutations [14,37]. Interestingly, except these 11 patients, all the other TVHTG patients had LPL activities similar to control subjects, with several high LPL values, as previously reported by Coca-Prieto [14]. As expected, our 4 patients with heterozygous LPL mutations were identified in the group of TVHTG patients with normal LPL activity except for one patient who additionally had neutralising anti LPL antibodies [27]. Surprisingly, LPL activity has been poorly documented in heterozygous *LPL* mutation carriers with a history of type V hyperlipidemia. According with our findings, Surendram [8] recently described 2 *LPL* and 1 *GPIHBP1* new heterozygous missense mutations with normal LPL activity. All these findings are consistent with the knowledge that under dietetic conditions, patients with heterozygous LPL mutations have normal or mildly increased TG levels due to sufficient residual LPL activity [38]. Interestingly, LPL activity levels in our patients with *APOA5* mutations were highly heterogeneous. Normal activity was found in one patient with a homozygous missense *APOA5* mutation (p.R343C) predicted to alter apoAV function. A very low LPL activity was found in 3 diabetic patients compound heterozygous for *APOA5* p.Q139X mutation and *APOA5* signal peptide p.S19W polymorphism. These patients were previously reported as having a very low activity using LPL activity emulsion method [24]. Finally, LPL activity was normal for 5 patients with other heterozygous deleterious *APOA5* mutations combined with the heterozygous p.S19W polymorphism. It remains possible that these variants are present on the same haplotype, leaving a functional *APOA5* allele in some of these patients. The discrepancy of LPL activity in *APOA5* deficient patients underlines the complexity of the phenotype and the necessity to have access to LPL activity in order to understand the high phenotypic variability of these patients. Occurrence of transient severe LPL deficiency might correspond to complex and not yet fully elucidated gene-gene interactions or gene-environment interactions.

In TVHTG patients, over half did not harbour any genetic mutation in *LPL*, *APOA5*, *APOC2*, *GPIHBP1*, *APOE*: 34% had only minor *APOA5* or *GPIHBP1* defects and 26% were not found to harbour any variants in candidate genes. This distribution is in complete accordance with Surendram [8] who had 47% HTG patients without mutations in candidate genes (26% with only polymorphism and 21% with no variants) and reports from other teams [7]. However LPL activity was not systematically reported in these series. Our new method identified 2 TVHTG patients with an apparent autosomal dominant transmission, a very low LPL activity, no deleterious mutation in any usual candidate genes (including *LMF1*) and no evidence for autoimmunity (Figure 3, lane 1).

These findings illustrate the importance of accurate routine LPL activity assessment in order to decipher the phenotype of TVHTG patients. The discrepancy between hyperchylomicronemia and normal LPL activities raises the question of the role of additional regulators of heparin releasable LPL and/or molecular mechanisms leading to transient LPL deficiencies. Although less decisive than molecular genetic testing for etiologic diagnosis, the finding of low LPL activity in patients without mutation or conversely very high activity in patients supposed to have major genetic defect, raise some major questions about the regulation of TG metabolism and bring valuable information for diagnosis. Due to its robustness and its excellent reproducibility, this new method using human VLDL as natural substrate provides a convenient tool in order to explore the underlying mechanisms of misunderstood hyperchylomicronemia.

Supporting Information

Data S1 Optimization of substrate.
(DOCX)

Figure S1 Optimization of the assay: triglycerides concentration in the mixture. Triglycerides concentration 25% (line); 50% (dotted line) Pool 23 (diamond); Pool 24 (circle) PHLA kinetics of 1 control plasma activity with 2 different VLDL substrates used at 2 TG concentrations (25 and 50%, i.e. TG 1.8 and 3.6 mmol/l).
(DOC)

Figure S2 PHLA measured with different triglycerides concentration substrate in the reaction. Patient 1 PHLA = 17.4 µmol/l/min (black line); Patient 2 PHLA = 49.2 µ–mol/l/min (grey line) 100% of activity was fixed with triglycerides in the mixture at 1.8 mmol/l.
(DOC)

Table S1 Lipids and apoproteins of the VLDL substrates (n = 40 different pools of VLDL).
(DOC)

Table S2 Genetic variants identified in candidate genes in TVHTG patients.
(DOC)

Acknowledgments

We would like to thank Pr Gilles Rioufol, Pr Gérard Finet, Dr François Sassolas, and Pierre-Valentin Laffitte for their contribution in control recruitment, and Dr Bénédicte Mousson de Camaret and Marjorie Dumoux for technical assistance.

Author Contributions

Conceived and designed the experiments: MDF CM SC ML PM AS. Performed the experiments: OM MB MD MM AN VPD. Analyzed the data: MDF CM SC OM MB MD MM AN VPD. Contributed reagents/materials/analysis tools: OM MB MD MM AN VPD RV. Wrote the paper: MDF CM SC ML PM AS RV.

References

1. Eckel RH (1989) Lipoprotein lipase. A multifunctional enzyme relevant to common metabolic diseases. N Engl J Med 320: 1060–1068.
2. Olivecrona G, Olivecrona T (1995) Triglyceride lipases and atherosclerosis. Curr Opin Lipidol 6: 291–305.
3. Dallinga-Thie GM, Franssen R, Mooij HL, Visser ME, Hassing HC, et al. (2010) The metabolism of triglyceride-rich lipoproteins revisited: new players, new insight. Atherosclerosis 211: 1–8.
4. Davies BS, Beigneux AP, Fong LG, Young SG (2012) New wrinkles in lipoprotein lipase biology. Curr Opin Lipidol 23: 35–42.
5. Hayden MR, Ma Y (1992) Molecular genetics of human lipoprotein lipase deficiency. Mol Cell Biochem 113: 171–176.
6. Johansen CT, Kathiresan S, Hegele RA (2011) Genetic determinants of plasma triglycerides. J Lipid Res 52: 189–206.
7. Gotoda T, Shirai K, Ohta T, Kobayashi J, Yokoyama S, et al. (2012) Diagnosis and management of type I and type V hyperlipoproteinemia. J Atheroscler Thromb 19: 1–12.
8. Surendran RP, Visser ME, Heemelaar S, Wang J, Peter J, et al. (2012) Mutations in LPL, APOC2, APOA5, GPIHBP1 and LMF1 in patients with severe hypertriglyceridaemia. J Intern Med 272: 185–196.
9. Boberg J, Carlson LA (1964) Determination of Heparin-Induced Lipoprotein Lipase Activity in Human Plasma. Clin Chim Acta 10: 420–427.
10. Biale Y, Shafrir E (1969) Lipolytic activity toward tri- and monoglycerides in postheparin plasma. Clin Chim Acta 23: 413–419.
11. Nilsson-Ehle P, Tornquist H, Belfrage P (1972) Rapid determination of lipoprotein lipase activity in human adipose tissue. Clin Chim Acta 1972: 383–390.
12. Henderson AD, Richmond W, Elkeles RS (1993) Hepatic and lipoprotein lipases selectively assayed in postheparin plasma. Clin Chem 39: 218–223.
13. Eriksson JW, Buren J, Svensson M, Olivecrona T, Olivecrona G (2003) Postprandial regulation of blood lipids and adipose tissue lipoprotein lipase in type 2 diabetes patients and healthy control subjects. Atherosclerosis 166: 359–367.
14. Coca-Prieto I, Valdivielso P, Olivecrona G, Ariza MJ, Rioja J, et al. (2009) Lipoprotein lipase activity and mass, apolipoprotein C-II mass and polymorphisms of apolipoproteins E and A5 in subjects with prior acute hypertriglyceridaemic pancreatitis. BMC Gastroenterol 9: 46.
15. Basu D, Manjur J, Jin W (2011) Determination of lipoprotein lipase activity using a novel fluorescent lipase assay. J Lipid Res 52: 826–832.
16. McFarland JT, Rojas C (1986) 1,2-Dipalmitoyl-3-β-2-furylacryloyltriacylglycerol: a chromophoric substrate for lipoprotein lipase. Biochem Biophys Acta 876: 438–449.
17. Dousset N, Negre A, Salvayre R, Rogalle P, Dang QQ, et al. (1988) Use of a fluorescent radiolabeled triacylglycerol as a substrate for lipoprotein lipase and hepatic triglyceride lipase. Lipids 23: 605–608.
18. Rawyler A, Siegenthaler PA (1989) A single and continuous spectrophotometric assay for various lipolytic enzymes, using natural, non-labelled lipid substrates. Biochim Biophys Acta 1004: 337–344.
19. Wicher I, Sattler W, Ibovnik A, Kostner GM, Zechner R, et al. (1996) Quantification of lipoprotein lipase (LPL) by dissociation-enhanced lanthanide fluorescence immunoassay. Comparison of immunoreactivity of LPL mass and enzyme activity of LPL. J Immunol Methods 192: 1–11.
20. Panteghini M, Bonora R, Pagani F (2001) Measurement of pancreatic lipase activity in serum by a kinetic colorimetric assay using a new chromogenic substrate. Ann Clin Biochem 38: 365–370.
21. Hendrickson HS (1994) Fluorescence-based assays of lipases, phospholipases, and other lipolytic enzymes. Anal Biochem 219: 1–8.
22. Baginsky ML, Brown WV (1979) A new method for the measurement of lipoprotein lipase in postheparin plasma using sodium dodecyl sulfate for the inactivation of hepatic triglyceride lipase. J Lipid Res 20: 548–556.
23. Marcais C, Bernard S, Merlin M, Ulhmann M, Mestre B, et al. (2000) Severe hypertriglyceridaemia in Type II diabetes: involvement of apoC-III Sst-I polymorphism, LPL mutations and apo E3 deficiency. Diabetologia 43: 1346–1352.
24. Marcais C, Verges B, Charriere S, Pruneta V, Merlin M, et al. (2005) Apoa5 Q139X truncation predisposes to late-onset hyperchylomicronemia due to lipoprotein lipase impairment. J Clin Invest 115: 2862–2869.
25. Charriere S, Peretti N, Bernard S, Di Filippo M, Sassolas A, et al. (2011) GPIHBP1 C89F neomutation and hydrophobic C-terminal domain G175R mutation in two pedigrees with severe hyperchylomicronemia. J Clin Endocrinol Metab 96: E1675–1679.
26. Charriere S, Cugnet C, Guitard M, Bernard S, Groisne L, et al. (2009) Modulation of phenotypic expression of APOA5 Q97X and L242P mutations. Atherosclerosis 207: 150–156.
27. Pruneta-Deloche V, Marcais C, Perrot L, Sassolas A, Delay M, et al. (2005) Combination of circulating antilipoprotein lipase (Anti-LPL) antibody and heterozygous S172 fsX179 mutation of LPL gene leading to chronic hyperchylomicronemia. J Clin Endocrinol Metab 90: 3995–3998.
28. Schotz MC, Garfinkel AS, Huebotter RJ, Stewart JE (1970) A rapid assay for lipoprotein lipase. J Lipid Res 11: 68-69.
29. Huttunen JK, Ehnholm C, Kinnunen PK, Nikkila EA (1975) An immunochemical method for the selective measurement of two triglyceride lipases in human postheparin plasma. Clin Chim Acta 63: 335–347.
30. Nilsson-Ehle P, Schotz MC (1976) A stable, radioactive substrate emulsion for assay of lipoprotein lipase. J Lipid Res 17: 536-541.
31. Nicoll A, Lewis B (1980) Evaluation of the roles of lipoprotein lipase and hepatic lipase in lipoprotein metabolism: in vivo and in vitro studies in man. Eur J Clin Invest 10: 487–495.
32. Posner I, Wang CS, McConathy WJ (1983) Kinetics of bovine milk lipoprotein lipase and the mechanism of enzyme activation by apolipoprotein C-II. Biochemistry 22: 4041–4047.
33. Blache D, Bouthillier D, Davignon J (1983) Simple, reproducible procedure for selective measurement of lipoprotein lipase and hepatic lipase. Clin Chem 29: 154–158.
34. Henriksen FL, Petersen PH, Beck-Nielsen H, Horder M (2001) Calibration, specificity and trueness of a postheparin plasma lipoprotein lipase assay. Clin Chem Lab Med 39: 263–269.
35. Imamura S, Kobayashi J, Nakajima K, Sakasegawa S, Nohara A, et al. (2008) A novel method for measuring human lipoprotein lipase and hepatic lipase activities in postheparin plasma. J Lipid Res 49: 1431–1437.
36. Babirak SP, Iverius PH, Fujimoto WY, Brunzell JD (1989) Detection and characterization of the heterozygote state for lipoprotein lipase deficiency. Arteriosclerosis 9: 326–334.
37. Rahalkar AR, Giffen F, Har B, Ho J, Morrison KM, et al. (2009) Novel LPL mutations associated with lipoprotein lipase deficiency: two case reports and a literature review. Can J Physiol Pharmacol 87: 151–160.
38. Ooi EM, Russell BS, Olson E, Sun SZ, Diffenderfer MR, et al. (2012) Apolipoprotein B-100-containing lipoprotein metabolism in subjects with lipoprotein lipase gene mutations. Arterioscler Thromb Vasc Biol 32: 459–466.
